# Further advances in knowledge on the role of the private sector in health systems

**DOI:** 10.1093/heapol/czu018

**Published:** 2014-07-07

**Authors:** Birger C Forsberg, Dominic Montagu

**Affiliations:** ^1^Department of Public Health Sciences, Karolinska Institutet, Stockholm S-171 76, Sweden, ^2^University of California, San Francisco, CA, USA

**Keywords:** Private sector, health system, low- and middle income countries, private care, public-private partnerships

Nearly 50% of total expenditure on health in low- and middle-income countries is private, rising to ∼70% in countries such as Pakistan, Liberia, India and Myanmar (World Health Organization 2011). A large proportion of this expenditure goes to private providers of health-related goods and services. Awareness of this, the scale and complexity of private health service provision and the magnitude of the regulatory challenges it presents, has been growing for a decade. As a response to the need for improved understanding of the role of the private sector in health systems, interested experts in health and economics have been working together in an informal consortium since 2008 to create a platform for continued growth of the knowledge on this subject. The consortium includes academic institutions and organizations with engagement in health advancement in low- and middle-income countries. Since 2009, the consortium has arranged a symposium every second year before the World Congress of the International Health Economics Association. The symposia are envisaged as a dialogue between researchers, international organizations and policy makers with a goal of moving forward the discussion on the role of the private sector in health systems in low- and middle-income countries. For all sessions, authors are encouraged to consider the relevance and implications of their findings as they apply to health systems in low- and middle-income countries.

Selected papers from the first symposium in this series were published in a supplement to *Health Policy and Planning* in 2011 (*Health Policy Plan* 2011).

This new supplement to *Health Policy and Planning* is an outcome of the Second Symposium on the Role of the Private Sector in Health Systems held in Toronto on 9 July 2011. The symposium invited researchers and policy makers around the world to participate with presentations on five themes central to the scientific development of understanding on the role of the private sector in health systems.

- Paying for private care: the private sector and health financing.
- Good, bad, or indistinguishable: quality of care in the private sector.
- Human resources for health and the private sector.
- The private sector role in the overall health system: policy issues in the private sector.
- Juxtaposing private health care in rich and poor countries.

A number of strong presentations were given at the symposium (The Private Sector in Health 2011).

Authors of the best presentations were invited to submit papers to *Health Policy and Planning* for consideration. Papers submitted were subjected to the usual meticulous review applied by the journal. In the end, five papers were accepted for publication. The papers are an illustration of the improved understanding of the private sector that the symposia have contributed to. Here follows a brief on each of the articles in the supplement.

Levey and Wang present a study of HIV counselling and testing (VCT) services in the public and private sectors in Zambia. The study used five primary data collection methods to gauge quality of VCT services. Over 400 clients and 87 facility managers were interviewed from almost 90 facilities. The study showed concerning levels of underperformance in VCT services across the sectors. Less than one-third of clients received counselling on reducing number of sexual partners and only ∼5% of clients received counselling about disclosing test results to partners. The private for-profit providers performed equally or sometimes better than other sectors in spite of the fact that this sector was not adequately integrated into the Zambian national response to HIV. Still, the overriding problem was that too few clients in general received adequate counseling. The authors conclude that in a generalized HIV epidemic like in Zambia risk-reduction methods and discussion should be a main focus of pre-test and post-test counselling. It can be concluded from the paper that the private sector has an important role to play in reaching the target groups for VCT.

Gautham *et al.* have studied informal health providers in North and South India. Rural households in India rely extensively on informal biomedical providers, who lack valid medical qualifications. The study reports on the education, knowledge, practices and relationships of informal providers (IPs) in two very different districts: Tehri Garhwal in Uttarakhand (northern India) and Guntur in Andhra Pradesh (southern India). In the districts, IPs were mapped and interviewed about market practices, relationships with the formal sector, and their knowledge of protocol-based management of fever, diarrhoea and respiratory conditions. Provider–patient interactions were observed. The study showed that IPs in the two districts had very different educational backgrounds—more years of schooling followed by various informal diplomas in Tehri and more apprenticeships in Guntur—yet their knowledge of management of the three conditions was similar and reasonably high.

IPs in Tehri were mostly clinic-based and dispensed a blend of allopathic and indigenous drugs. IPs in Guntur mostly provided door-to-door services and prescribed and dispensed mainly allopathic drugs. In Guntur, formal private doctors were important referral providers (with commissions) and source of new knowledge for IPs. At both sites, IPs prescribed inappropriate drugs, but the use of injections and antibiotics was higher in Guntur. Guntur IPs were well organized in state and block level associations that had successfully lobbied for a state government registration and training for themselves.

The study shows that IPs are firmly established in rural India but their role has grown and evolved differently in different market settings. The authors conclude that interventions need to be tailored differently keeping in view unique features of each provider group.

Hotchkiss, Godha and Do look at expansion in the private sector provision of institutional delivery services and horizontal equity in Nepal and Bangladesh. The researchers explore if use of appropriate maternal healthcare services can be increased by encouraging the expansion of the role of the private sector, and whether doing so will significantly increase inequity. The study is based on multiple rounds of nationally representative household survey data collected in Nepal and Bangladesh from 1996 to 2011. The methodology involves estimating a concentration index for each survey to assess changes in wealth-related inequity in the use of institutional delivery assistance over time. The results of the study suggest that the expansion of private sector supply of institutional-based delivery services in Nepal and Bangladesh has not led to increased horizontal inequity. In fact, in both countries, inequity is shown to have decreased over the study period. The study findings also suggest that the provision of government delivery services to the poor protects against increased wealth-related inequity in service use.

Kanya *et al.* have studied the coverage of facility deliveries by a maternal health voucher programme in Southwestern Uganda and examined whether such coverage is correlated with district-level characteristics such as poverty density and the number of contracted facilities. The results show that:

- the programme paid for 38% of estimated deliveries among the poor population in the targeted districts,
- there was a significant negative correlation between the poverty density in a district and proportions of births to poor women that were covered by the programme and
- improving coverage of health facility deliveries for poor women is dependent upon increasing the sales and redemption rates of vouchers.

The findings suggest that to the extent that the programme stimulated demand for Safe Motherhood services by new users, it has the potential of increasing facility-based births among poor women in the region. In addition, the significant negative correlation between the poverty density and the proportions of facility-based births to poor women that are covered by the voucher programme suggests that there is need to increase both voucher sales and the rate of redemption to improve coverage in districts with high levels of poverty.

Pomeroy, Koblinski and Alva explore who gives birth in private facilities in Asia through a review of data from six countries. The authors use Demographic and Health Survey data to examine trends in growth of delivery care provided by private facilities and describe who is using the private sector within the healthcare system. Results show a significant trend towards greater use of private sector delivery care over the last decade. Wealth and education are related to private sector delivery care in about half of the countries, but are not as universally related to use as one might expect. A previous private facility birth predicted repeat private facility use across nearly all countries in this study. In Cambodia and India, primiparity also predicted private facility use. More in-depth work is needed to truly understand the behaviour of the private sector in these countries; the results warn against making generalizations across countries about private sector delivery care.

Taken together, these five papers provide a snapshot of both current research and current issues in global health where the importance of the private sector is becoming more apparent and is increasingly recognized as critical to many of the major health issues prioritized by both national governments and global health initiatives. Three of these five papers deal with issues related to private provision of facility-based delivery services. As the global trend towards facility deliveries accelerates, government facilities in many countries have failed to keep pace with the increase in demand. One result of this has been an increase in the number of private delivery centres, and a growing acceptance that expanding national health insurance programmes (e.g. in Ghana, India and the Philippines) can and should reimburse private facilities at equal rates to public facilities.

The history of European health care shows clearly that there are many ways to achieve widely shared equitable health services. Whether funding comes primarily through general taxation (as in the UK) or through mandatory social health insurance (as in German and Holland), seems to matter little or not at all so long as broader policy and economic systems function well. But regardless of funding, the experience of Europe, and of The Organisation for Economic Co-operation and Development (OECD) more broadly, makes clear that active financing of, and engagement with, private providers of health care—as in the case of the private delivery facilities highlighted in these papers—is nearly always an integral and positive component of the larger health system delivery functions.

Where formal systems break down—with IPs in India, or VCT quality in Zambia—financing matters less than national systems for regulatory oversight and quality assurance. As these papers show, the need for that remains high in many countries.

That stewardship of the private sector can only follow from a greater understanding of, and appreciation of, the role that private care has within well functioning health systems. The bi-annual Symposia on the Private Sector in Health, and growing research in this field, is a reason for optimism that this understanding and attention is indeed becoming stronger. These five papers therefore are part of a larger movement towards an inclusive, and holistic, understanding of health care financing and delivery that gives us hope for the future of health planning and research.

*Conflict of interest statement*: None declared.
